# Human Keratinocyte UVB-Protective Effects of a Low Molecular Weight Fucoidan from *Sargassum horneri* Purified by Step Gradient Ethanol Precipitation

**DOI:** 10.3390/antiox9040340

**Published:** 2020-04-21

**Authors:** Ilekuttige Priyan Shanura Fernando, Mawalle Kankanamge Hasitha Madhawa Dias, Disanayaka Mudiyanselage Dinesh Madusanka, Eui Jeong Han, Min Ju Kim, You-Jin Jeon, Kyounghoon Lee, Sun Hee Cheong, Young Seok Han, Sang Rul Park, Ginnae Ahn

**Affiliations:** 1 Department of Marine Bio-Food Sciences, Chonnam National University, Yeosu 59626, Korea; shanurabru@gmail.com (I.P.S.F.); sunny3843@chonnam.ac.kr (S.H.C.); 2 Department of Food Technology and Nutrition, Chonnam National University, Yeosu 59626, Korea; hasithadiasm17636@gmail.com (M.K.H.M.D.); dmdmadusanka88@gmail.com (D.M.D.M.); iosu5772@naver.com (E.J.H.); mijoo92@naver.com (M.J.K.); 3 Marine Science Institute, Jeju National University, Jeju Self-Governing Province 63333, Korea; youjin2014@gmail.com; 4 Division of Fisheries Science, Chonnam National University, Yeosu 59626, Korea; ricky1106@naver.com; 5 Neo Environmental Business Co., Daewoo Technopark, Doyak-ro, Bucheon 14523, Korea; hanulva@neoenbiz.com; 6 Estuarine & Coastal Ecology Laboratory, Department of Marine Life Sciences, Jeju National University, Jeju 63243, Korea; srpark@jejunu.ac.kr

**Keywords:** oxidative stress, ultraviolet B, gradient ethanol precipitation, fucoidan, HaCaT keratinocyte, heme oxygenase-1, nutricosmetic

## Abstract

Ultraviolet B (UVB) radiation-induced oxidative skin cell damage is a major cause of photoaging. In the present study, a low molecular weight fucoidan fraction (SHC4) was obtained from *Sargassum horneri* by Celluclast-assisted extraction, followed by step gradient ethanol precipitation. The protective effect of SHC4 was investigated in human keratinocytes against UVB-induced oxidative stress. The purified fucoidan was characterized by Fourier-transform infrared spectroscopy (FTIR), ^1^H nuclear magnetic resonance (NMR), agarose gel-based molecular weight analysis and monosaccharide composition analysis. SHC4 had a mean molecular weight of 60 kDa, with 37.43% fucose and 28.01 ± 0.50% sulfate content. The structure was mainly composed of α-L-Fucp-(1→4) linked fucose units. SHC4 treatment dose-dependently reduced intracellular reactive oxygen species (ROS) levels and increased the cell viability of UVB exposed HaCaT keratinocytes. Moreover, SHC4 dose-dependently inhibited UVB-induced apoptotic body formation, sub-G_1_ accumulation of cells and DNA damage. Inhibition of apoptosis was mediated via the mitochondria-mediated pathway, re-establishing the loss of mitochondrial membrane potential. The UVB protective effect of SHC4 was facilitated by enhancing intracellular antioxidant defense via nuclear factor erythroid 2–related factor 2 (Nrf2)/heme oxygenase-1 (HO-1) signaling. Further studies may promote the use of SHC4 as an active ingredient in cosmetics and nutricosmetics.

## 1. Introduction

Ultraviolet B (UVB)-irradiation (280–320 nm) primarily induces cell damage by augmenting intracellular reactive oxygen species (ROS) levels. The radiation easily penetrates the stratum corneum, where it increases the risk of photoaging, with symptoms characterized by thickening of the epidermis, discoloration, skin wrinkling, loss of elasticity and skin cell growth retardation [1]. Recent developments in marine bioresource technology have substantiated the therapeutic potential of marine natural products to be used as cosmeceuticals, functional foods, new drugs and drug leads. Fucoidan is a fucose-rich sulfated polysaccharide found in brown algae that is renowned for its versatile biologic activities. High molecular weight fucoidans closely resemble the structure of heparin and are effective anticoagulants [2]. Nevertheless, low molecular weight fucoidans are reported to possess antioxidant, anti-inflammatory, anticancer and anti-microbial activities [3,4]. Fucoidan mainly contains α-1,3 and α-1,4-linked fucose units with sulfate ester substituents at the 2, 3 and/or 4 positions. However, the chains could also contain monosaccharides such as glucose, mannose, xylose, arabinose and hexuronic acid. The monomer composition, connectivity, branching, sulfation pattern, and molecular weight are species-related [2]. The conventional method of refining fucoidan involves hot water extraction of dry algal powder under mildly acidic pH, followed by ethanol precipitation. The precipitate undergoes additional fractionation via ultrafiltration, size exclusion or anion exchange chromatography. This method lacks efficiency based on the low extraction yield, convenience, time consumption and selectivity over molecular weights.

As fucoidan is mainly localized to the cell wall of brown algae, degradation of the cell wall using enzymes is a well-planned extraction strategy conserving polymer integrity, and thereby its biofunctional properties. The use of enzymes, including Celluclast, Termamyl, Ultraflo, Amyloglucosidase, and Viscozyme, is advisable to obtain fucoidan whose biofunctional properties are preserved [5]. Gradient alcohol precipitation is a fractionation method used for fractionating polysaccharides with gradually narrowed down molecular weight distributions [6]. It is a relatively inexpensive and simple method compared to ultrafiltration and chromatography. The precipitation is achieved by incorporating organic solvents such as methanol, ethanol, isopropanol, 1-butanol and acetone or concentrated inorganic salts such as ammonium sulfate ((NH_4_)_2_SO_4_) and polymerizing agents such as polyethylene glycol (PEG). The use of this method has been previously reported for fractionating fucoidan [7]. The present study was undertaken as a part of a project to investigate possible industrial uses of *S. horneri* as a sustainable approach for managing its large biomass. The extraction of fucoidan enriched crude polysaccharides followed an optimized green approach using enzymes, while the fractionation followed a step gradient ethanol precipitation.

## 2. Materials and Methods

Fucoidan standard, KBr (FTIR grade), deuterium oxide, 2′,7′-dichlorodihydrofluorescein diacetate (DCFH2-DA) and 3-(4,5-dimethylthiazol-2-yl)-2,5-diphenyltetrazolium bromide (MTT), o-Toluidine blue, trifluoroacetic acid and 2-mercaptoethanolwere purchased from Sigma-Aldrich (St. Louis, MO, USA). Celluclast was obtained from Novozyme Co. (Bagsvaerd, Denmark). Chloroform, methanol and ethanol were of analytical grade. Dulbecco’s Modified Eagle Medium (DMEM), fetal bovine serum (FBS) and penicillin/streptomycin mixture were purchased from GIBCO INC., (Grand Island, NY, USA). Primary and ary antibodies were purchased from Cell Signalling Technology, Inc. (Beverly, MA, USA) and Santa Cruz Biotechnology (Santa Cruz, CA, USA).

### 2.1. Preparation of Fucoidan Fraction

#### 2.1.1. Extraction of Fucoidan Enriched Polysaccharide

Washed and dried *S. horneri* samples collected off Jeju coast were provided to us by Seojin Biotech Company limited. The samples were pulverized using an MF 10 basic, IKA microfine grinder (Werke, Germany). Depigmentation was carried out using a solvent system of chloroform and methanol 1:1. Next, the dried powder was soaked in a solution of ethanol containing 10% formaldehyde for 3 h at 40 °C. The dried powder was washed twice with 80% ethanol. After evaporating off any remaining solvent, the sample powder was suspended in 5 L of deionized water at a 1:10 (kg/L) ratio. The pH was adjusted to 4.5 by adding diluted HCl while equilibrating at 50 °C in a shaking incubator for 1 h. Celluclast was added at a 0.5% sample ratio and kept for 8 h under continuous agitation at 50 °C. The mixture was filtered through a muslin cloth. Celluclast was heat-denatured at 100 °C for 10 min. The extract was neutralized at room temperature by adding diluted NaOH and centrifuged at 5000× *g* for 20 min to remove unfiltered particles. The supernatant (4.5 L) was frozen and lyophilized to reduce the volume to 1 L.

#### 2.1.2. Step Gradient Ethanol Precipitation

**Figure 1:**
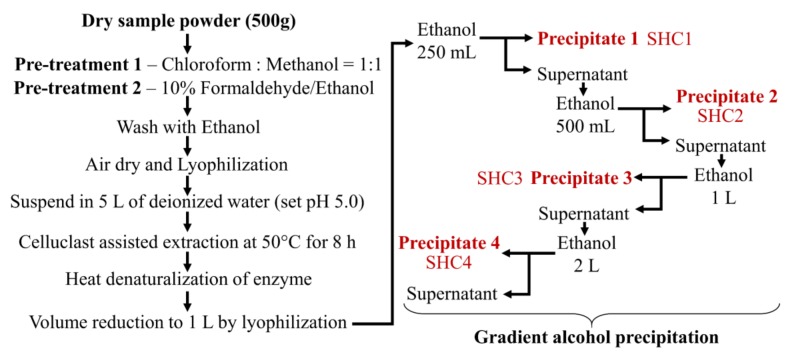
The procedure of sample pretreatment, enzyme-assisted extraction, and fractionation by gradient ethanol precipitation.

The ratio of ethanol was determined following optimization studies. As the first step in gradient ethanol precipitation, 250 mL of ethanol was gently added to 1 L of the extract while stirring. The mixture was incubated at 4 °C for 12 h, allowing it to equilibrate while precipitating the polysaccharides (Figure 1). After, the mixture was centrifuged at 5000× *g* for 20 min at 4 °C to obtain the first precipitate designated as SHC1. Sequentially the second, third and fourth precipitates were collected by, respectively adding 500 mL, 1 L and 2 L of ethanol to the supernatant after each precipitation step. All precipitates were dually washed with 95% ethanol (homogenization) and centrifuged to recover the polymer. Finally, the precipitates were dissolved in deionized water and dialyzed using 3.5-kD molecular weight cutoff dialysis membranes (Spectra/Por, Los Angeles, CA, USA). Polysaccharide fractions were lyophilized and stored at −20 °C for proceeding experiments.

### 2.2. Analysis of Molecular Weight (MW) Distribution

Approximate molecular weight distribution, homogeneity, and separation efficiency of the polysaccharide fractions were analyzed by an agarose gel electrophoresis method [3]. Briefly, markers and samples (1 mg mL^−1^) were electrophoresed in 1% agarose gels in Tris-Borate-EDTA running buffer (pH 8.3) at 100 V for 20 min. The gel was stained with 0.02% toluidine blue and 0.5% Triton X-100 in 3% acetic acid and de-stained with 3% acetic acid.

### 2.3. Fourier-Transform Infrared Spectroscopy (FTIR) and Monosaccharide Composition Analysis

Polysaccharide powders were cast into KBr pellets and analyzed by a VERTEX 70v FTIR spectrometer (Bruker, Germany) [3]. For the monosaccharide composition analysis, polysaccharides were hydrolyzed with 4 M of trifluoroacetic acid and separated on a CarboPac PA1 column integrated to a Dionex ED50 Detector (HPAEC-PAD) (Dionex, Sunnyvale, CA, USA). A standardized monosaccharide mixture was used as the reference standard [3].

### 2.4. H^1^ Nuclear Magnetic Resonance (NMR) Analysis

The selected polysaccharide fraction, SHC4, was deuterium exchanged by co-lyophilizing with deuterium oxide, dissolved in deuterium oxide, and analyzed by a JNM-ECX400, 400 MHz spectrometer (JEOL, Tokyo, Japan).

### 2.5. In Vivo Cell Culture

#### 2.5.1. Cell Maintenance

HaCaT keratinocytes (Korean Cell Line Bank, Seoul, Korea) were maintained in DMEM containing 10% FBS and 1% penicillin-streptomycin. Cells were sub-cultured once every two days. Exponentially growing cells were used for seeding (2 × 10^5^ cells mL^−1^). After 24 h, wells were treated with different sample concentrations and further incubated for 24 h. Cytotoxicity was measured by MTT assay. Briefly, cells were incubated with MTT for 4 h, dissolved in dimethyl sulfoxide (DMSO), and the absorbance readings were taken at 540 nm using a SpectraMax^®^ M2 system (Molecular Devices, Sunnyvale, CA, USA).

#### 2.5.2. UVB Exposure and Oxidative Stress Analysis

HaCaT keratinocytes were incubated for 2 h with different concentrations of samples. The cell culture media was withdrawn from the wells, once washed, and then filled with PBS. Wells except the control were exposed to UVB (50 mJ cm^−2^) by a UVP CL-1000L ultraviolet cross-linker (Upland, CA, USA). Immediately after the irradiation, PBS was withdrawn, once washed, and replaced with serum-free culture media. Intracellular ROS levels were measured after incubating for an hour (DCFH2-DA assay) and cell viability was measured by MTT assay after incubating for 24 h [1]. SHC4 that indicated superior antioxidant and cytoprotective effects were further verified by DCFH2-DA staining of cells and analysis by fluorescence microscopy (EVOS FL Auto 2 Imaging, ThermoFisher Scientific, CA, USA) and flow cytometry (CytoFLEX, Beckman Coulter, PA, USA) based on methods described in our previous publications [8].

#### 2.5.3. Analysis of Nuclear Morphology

Cells were stained with either Hoechst 33342 or mixture of acridine orange and ethidium bromide according to the method described in our previous publication [9]. Nuclear morphologies were visualized on an EVOS FL Auto 2 Imaging microscope (Thermo-Fisher, Waltham, MA, USA).

#### 2.5.4. Western Blot Analysis

HaCaT cells after initial sample treatment and UVB-exposure were used for the experiments following a 24 h incubation period. Western blot analysis was done following our previously reported method [10]. A nuclear and cytoplasmic extraction kit, NE-PER^®^ (Thermo Scientific, Rockford, IL, USA), was used to isolate cytosolic and nucleic proteins. Proteins were standardized using a BCA^TM^ protein assay kit (Pierce, Rockford, IL, USA). Electrophoresis was carried out using 10% SDS-polyacrylamide gels. SuperSignal™ West Femto Maximum Sensitivity Substrate (Thermo, Burlington, ON, Canada) was used for the detection of protein bands on a Core Bio DavinchChemi imaging system (Seoul, Korea).

#### 2.5.5. Cell Cycle Analysis

Harvested cells were permeabilized in 70% ethanol for 30 min, incubated in PBS containing EDTA, RNase and propidium iodide at 37 °C for 30 min, and analyzed by a Beckman Coulter CytoFLEX flow cytometer (Brea, CA, USA). A dual-region gating approach, initial FS vs. SS to remove derbies and FL3-area vs. FL3-hight to eliminate doublets, was applied. Sub-G_1_ apoptotic cell population percentages were used for data compression.

#### 2.5.6. JC-1 Assay

Mitochondria membrane potential was ratiometrically measured by flow cytometry using MitoProbe JC-1 Assay Kit (Thermo Fisher Scientific, Waltham, MA, USA) following the manufacturer’s instructions. Cultured cells were initially subjected to sample treatment and UVB-exposure. After 4 h, cells were harvested for the assay.

#### 2.5.7. Comet Assay

DNA damage in individual cells was evaluated by alkaline comet assay following the procedure outlined in our previous publication [9]. Briefly, cells were harvested and suspended in low-melting agarose at 37 °C and gently applied on the surface of agarose coated slides. The slides were overnight immersed in alkaline lysis buffer at 4 °C. Electrophoresis was performed at 4 °C in an alkaline running buffer. Finally, the slides were submerged in a chilled neutralization buffer and stained with ethidium bromide. Comet tails were pictured on an EVOS FL Auto 2 Imaging microscope and tail DNA contents were quantified using OpenComet plugin in NIH Image J software (US National Institutes of Health, Bethesda, MD, USA).

### 2.6. Statistical Analysis

The data from experiments are presented as mean ± standard error (SE). Statistical comparisons were carried out by one-way analysis of variance by Duncan’s test using PASW Statistics 19.0 software (SPSS, Chicago, IL, USA). The level of significance was set at * *p* < 0.05 and ** *p* < 0.01.

## 3. Results

### 3.1. Extraction Efficiency and Proximate Compositions

The enzyme (Celluclast)-assisted extraction yield of *S. horneri* powder was 20.30 ± 0.32%. Yields corresponding to precipitated polysaccharides during step gradient ethanol precipitation are provided in Table 1. The highest yield was observed from fraction SHC2, obtained by incorporating 500 mL of ethanol. The proximate chemical compositions of the subsequent fractions indicated a gradual increase in the degree of sulfation in polysaccharides. The polyphenol, protein and ash content were comparatively low in all fractions, demonstrating the efficiency of the procedure in obtaining sulfated polysaccharides.

### 3.2. Molecular Weights Distribution of Polysaccharide Fractions and Their Vibrational Spectra

**Figure 2:**
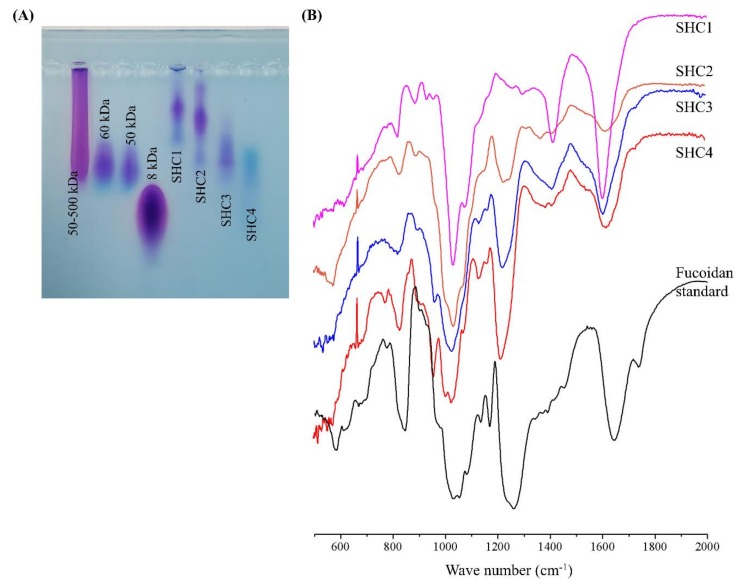
Characterization of polysaccharide fractions (SHC1–SHC4) obtained by step gradient ethanol precipitation. (**A**) Molecular weights (MW) distribution analysis of polysaccharide fractions compared to 50–500-kDa, 60-kDa, 50-kDa and 8-kDa MW standards and (**B**) vibrational spectra of polysaccharide fractions compared to commercial fucoidan standard.

Agarose gel electrophoresis has previously been used to estimate the weight distributions of anionic polysaccharides [11,12]. The heterogeneous nature of polysaccharides results in a weight distribution rather than a single band. As Figure 2A indicates, the molecular weight distributions of the polysaccharide fractions are gradually reduced in subsequent fractions. The approximate weight distributions were calculated using the Image J software based on the mean values of weight distributions of the molecular weight markers. Accordingly, the mean molecular weights of the fractions, SHC1, SHC2, SHC3 and SHC4, were estimated to be 230, 205, 90 and 60 kDa, respectively. The fingerprint region of the polysaccharide vibrational spectra (wavenumbers 2000–500 cm^−1^) is depicted in Figure 2B. The peak at 840 cm^−1^ represented C-O-S bending vibrations of axial sulfate substituents on the carbon at the 4 position (C-4) of fucose units. The prominent, broad peak between 1200–970 cm^−1^ occurred from the overlapping of peaks corresponding to C-C and C-O stretching vibrations of pyranoid rings and C-O-C stretching of glycosidic bonds, which are common to all polysaccharides. The major peak between 1220–1270 cm^−1^ and its minor encounter at 585 cm^−1^ were attributed to the O=S=O stretching vibrations of sulfates, which is a characteristic feature of sulfated polysaccharides such as fucoidans. The intense peak at the 1620 cm^−1^ region corresponds to the bending vibration of O-H moieties [13,14,15].

### 3.3. SHC4 Increased the Protective Effects against UVB-Induced Oxidative Stress

**Figure 3:**
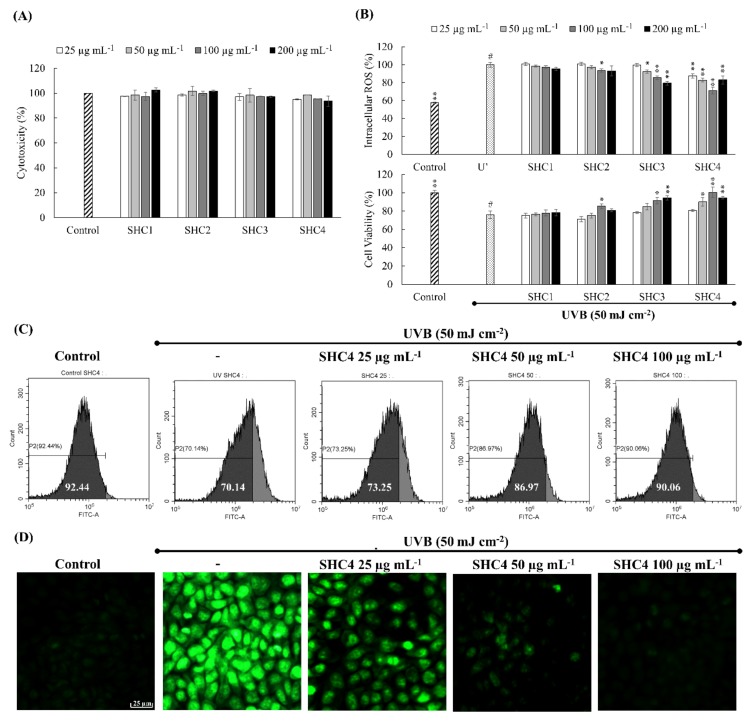
Protective effects of low molecular weight fucoidan fraction (SHC4) against ultraviolet B (UVB)-induced oxidative stress in HaCaT keratinocytes. (**A**) Cytotoxicity dose responses of polysaccharide fractions. (**B**) Analysis of intracellular reactive oxygen species (ROS) levels and cell viability after UVB exposure. Analysis of intracellular ROS levels by (**C**) flow cytometry and (**D**) fluorescence microscopy. HaCaT cells were treated with different doses of polysaccharide fractions for 2 h and exposed to UVB radiation. Measurement of intracellular ROS were performed 1 h after UVB exposure. Data represent the mean ± standard deviation of triplicate determinants (*n* = 3). * *p* < 0.05 and ** *p* < 0.01 are significantly different compared with group indicated by “#”.

The cytotoxicity of polysaccharide fractions within 25–200 µg mL^−1^ concentrations was examined prior to the evaluation of their bioactivities. None of the fractions indicated a significant decrease in cell proliferation within the tested concentration range (Figure 3A). Hence, the above concentrations were considered safe for the subsequent analysis of UVB protective effects. Based on DCFH2-DA and MTT assays, UVB irradiation significantly increased the intracellular ROS generation and inhibited cell proliferation (Figure 3B). All fucoidan fractions dose-dependently attenuated the effects of UVB irradiation. The best UVB protective effects were observed from the SHC4 fraction (25–100 µg mL^−1^): it significantly reduced UVB-induced intracellular ROS levels and increased cell proliferation. The 200 µg mL^−1^ concentration of SHC4 deviated from the dose-dependent response observed for the 25–100 µg mL^−1^ concentration range. Due to the increase seen in intracellular ROS levels and the affiliated inhibition of cell viability, the 200 µg mL^−1^ concentration was not used in subsequent analysis. Fluorescence-activated cell sorting (FACS) analysis using the DCFH2-DA fluoroprobe was considered reliable compared to the DCFH2-DA assay as it would omit the error caused by the decrease in fluorescence intensity due to cell death. As Figure 3C indicates, UV irradiation causes the peak to shift to higher intensity (Y-axis—FITC A) compared to the control. The effects were dose-dependently recovered with increasing concentrations of SHC4. Fluorescence microscopy analysis of DCFH2-DA fluoroprobe-treated cells indicated increased green fluorescence for ROS in UV-exposed cells compared to the control (Figure 3D), whereas the fluorescence was dose-dependently decreased with SHC4 treatment. Results of each ROS assay showed a moderate correlation, indicating the antioxidant effects of SHC4.

### 3.4. Characterization of SHC4 by NMR and Monosaccharide Composition Analysis

**Figure 4:**
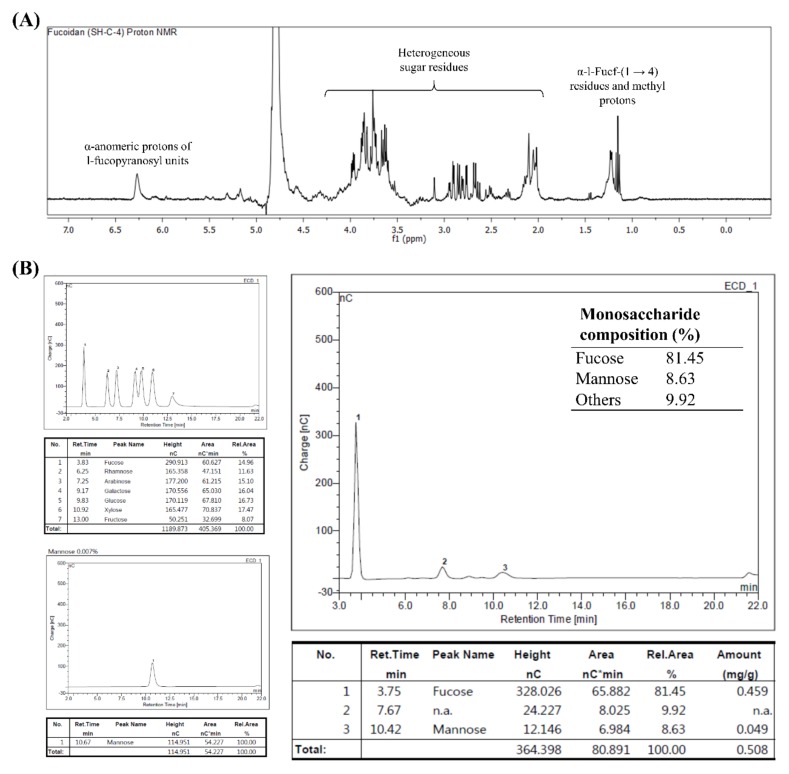
Characterization of fraction SHC4 by nuclear magnetic resonance (NMR) and monosaccharide composition analysis. SHC4 (**A**) ^1^H NMR spectrum and (**B**) monosaccharide composition analysis.

Integrated with FTIR spectra, monosaccharide composition analysis and NMR spectra provide reliable evidence to characterize fucoidans. The NMR spectrum of SHC4 (Figure 4A) was obtained with a relatively low resolution, mainly because of the heterogeneous nature of the polymer. However, we were able to identify some characteristic peaks in the spectrum, signifying fucoidans. The intense signals obtained between 1.1–1.2 ppm represented protons of methyl groups in fucopyranose. Based on previous records, the occurrence of peaks in the 1.1–1.2 ppm area is attributable to an α-L-Fucp-(1→4) connectivity pattern [16]. The proton signals between 2.0–2.2 ppm represent alcoholic protons [16], while signals between 3.5–4.0 ppm were attributed to H2-H6 sugar residues. The prominent signal at 4.6 ppm, 1[H] can be attributed to protons at the anomeric center of 3-linked d-galactopyranosyl residues [16,17]. The signal at 6.25 ppm indicates α-anomeric protons of l-fucopyranosyl units [15]. The obtained results were similar to findings recorded from other brown seaweeds [3,15,16,17]. HPAEC-PAD analysis of monosaccharide composition (Figure 4B) proposed higher fucose (81.45%) and mannose (8.63%) content, which suggested that SHC4 was a mannofucan.

### 3.5. SHC4 Reduced UVB-Induced Apoptotic Body Formation and DNA Damage

**Figure 5:**
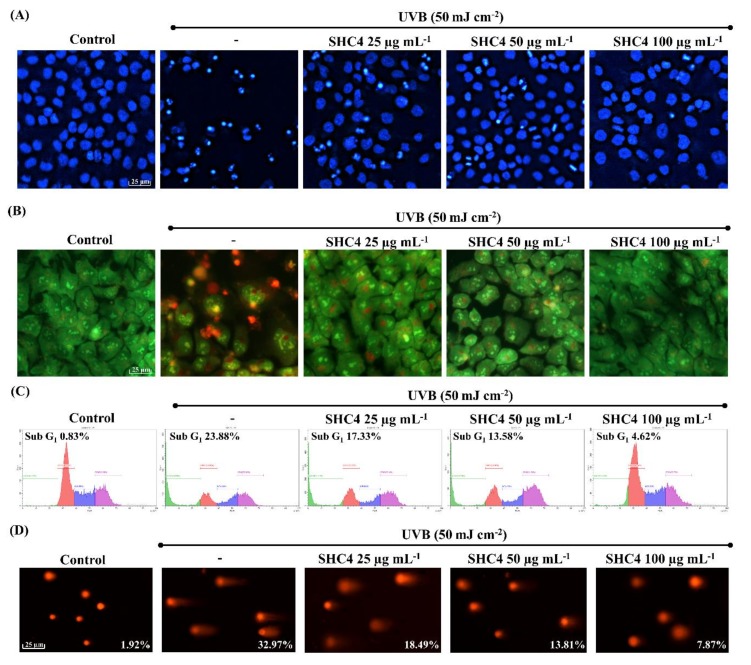
Effects of SHC4 on ultraviolet B (UVB)-induced apoptotic body formation and DNA damage. Nuclear morphology analysis of apoptotic body formation by (**A**) Hoechst 33342 and (**B**) nuclear double staining with ethidium bromide and acridine orange. (**C**) Cell cycle analysis of Sub-G_1_ apoptotic cell accumulation. (**D**) Analysis of DNA damage by comet assay. Comet-tail DNA contents were quantified using OpenComet plugin in ImageJ software. HaCaT cells were treated with different doses of polysaccharide fractions for 2 h and exposed to UVB radiation. Cells were harvested for experiments 1 h after UVB exposure. Repeatability of results was validated with three independent determinations (*n* = 3).

Cell death due to UVB-induced oxidative stress is reported to proceed via apoptosis [18]. Thus, we assessed apoptotic body formation and DNA damage. Hoechst 33,342 and nuclear double staining with ethidium bromide and acridine orange are the methods of choice for studying nuclear morphology. Hoechst 33,342 binds specifically to DNA, allowing the identification of apoptotic nuclei with DNA fragmentation and chromatin condensation [19]. A prompt increase of chromatin condensation and DNA fragmentation was seen in UVB-stimulated cells compared to the control (Figure 5A). The occurrence of orange-colored nuclear fragments in UVB-exposed cells upon nuclear double staining was indicative of late apoptotic events. SHC4 treatment dose-dependently suppressed the formation of apoptotic bodies, demonstrating its protective effects. According to Figure 5C, the increase in the Sub-G_1_ population (23.88%) further indicates UVB-induced apoptosis compared to non-irradiated controls (0.83%). SHC4 treatment dose-dependently reduced the Sub-G_1_ population. Additionally, DNA damage was examined by comet assay (Figure 5D). The increased tail DNA content was an indication of UVB-induced DNA damage. SHC4 treatment dose-dependently reduced UVB-induced cell damage.

### 3.6. SHC4 Exerted Its Protective Effects against UVB-Induced Oxidative Stress by Inhibiting Mitochondria-Mediated Apoptosis Signaling

**Figure 6:**
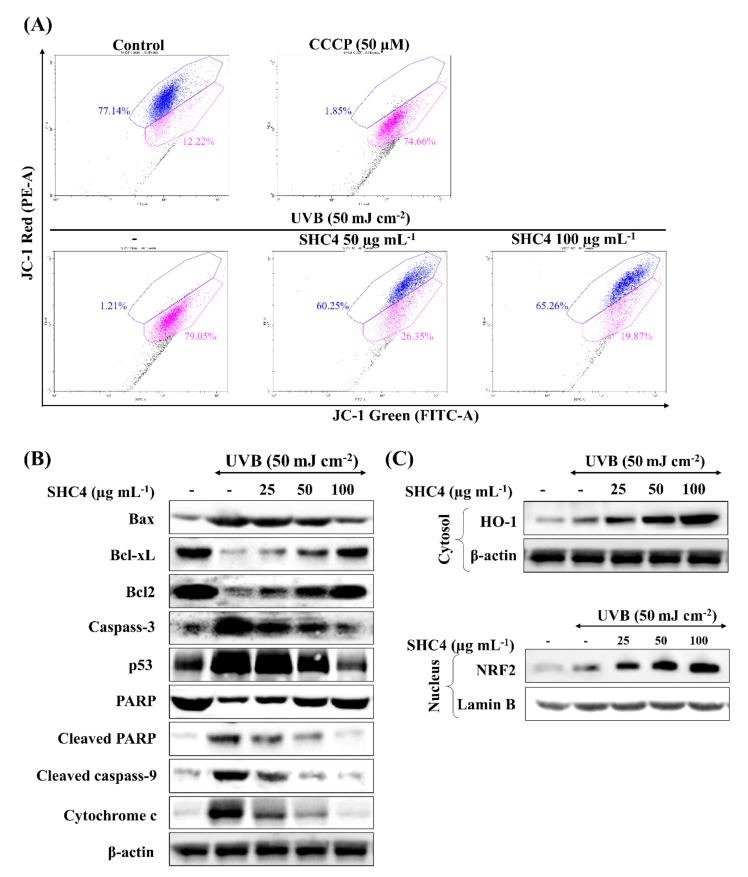
Western blot analysis of the protective effects of SHC4 against UVB-induced apoptosis mediators. Analysis of the UVB protective effects of SHC4 on (**A**) changes in mitochondria inner transmembrane potential by JC-1 assay, (**B**) mediation of mitochondria-mediated apoptotic pathway proteins against UVB-induced apoptosis and (**C**) effects on intracellular antioxidant enzymes. HaCaT cells were treated with different doses of SHC4 for 2 h and exposed to UVB radiation. Cells were, respectively harvested after 4 h and 24 h for the JC-1 assay and western blot analysis. Repeatability of results was validated with three independent determinations (*n* = 3).

The loss of the mitochondrial membrane potential due to pro- and anti-apoptotic Bcl-2 family proteins cause a subsequent discharge of caspase activators inducing apoptosis. The collapse of the mitochondrial inner transmembrane potential (ΔΨm) causes the opening of permeability transition pores resulting in an inward flux of water, which causes swelling and disruption of the outer membrane [20]. Based on a flow cytometry JC-1 assay (Figure 6A), a higher proportion of JC-1 aggregates with 530 nm emission (unhealthy mitochondria) were observed in UVB-exposed cells (78.77%), which was analogous to carbonyl cyanide m-chlorophenyl hydrazone (CCCP) treated cells. CCCP is a potent disruptor of the mitochondrial membrane potential. The membrane potential was dose-dependently attenuated following SCOC4 treatment, as evidenced by the increased emission at 590 nm and decreased emission at 530 nm. Key molecular mediators of the mitochondria-mediated apoptosis pathway were studied by western blot analysis (Figure 6B). An immediate increase was observed for BAX, caspases-3 and 9, p53, cleaved P poly (ADP-ribose) polymerase (PARP) and cytochrome C upon UVB-stimulation, whereas the anti-apoptotic proteins, Bcl-xL and Bcl2 levels were decreased. SHC4 dose-dependently attenuated levels of molecular mediators, exhibiting its protective effects. Intracellular antioxidant enzymes, such as heme oxygenase-1 (HO-1), glutathione peroxidase (GPx), catalase (CAT) and superoxide dismutase (SOD) play a crucial role in maintaining the redox balance in cells. Compounds that can upregulate the transcription of the above antioxidant enzymes offer protective effects against oxidative stress, which can lead to apoptosis [21]. According to Figure 6C, UVB exposure slightly increases Nrf2 levels in the nucleus as well as HO-1 levels in the cytosol. SHC4 treatment further induced Nrf2 and HO-1 levels in UVB exposed keratinocytes in a dose-dependent manner.

## 4. Discussion

The edible brown seaweed *Sargassum horneri* has long been acknowledged for its biofunctional effects. It is abundant along the Jeju island coasts in South Korea. Currently, the native *S. horneri* population is threatened by the massive invasion of *S. horneri* from the east coast of China [22]. This not only threatens the coastal ecosystem but also reduces the attraction for tourists. Climate changes have contributed to warmer temperatures and increased rainfall, which increase fertilizer runoff from paddy fields and the aquaculture industry, adding nutrients to coastal areas. The drastic change observed in *S. horneri* growth pattern is an example of how human activities alter the environmental balance. Other than clearing up the coasts, the utilization of *S. horneri* in the food industry and for obtaining functional ingredients such as polyphenols, fucoidan and alginic acid could be an effective and beneficial strategy.

Celluclast is a food-grade cellulase that hydrolyzes cell wall cellulose, resulting in a higher extraction yield of plant materials. The water-based extract under pH 4.5 may contain ions and polar metabolites such as phenols, soluble polysaccharides and proteins. Alternatively, extraction at acidic pH greatly reduces alginate contamination. Based on preliminary investigations, the addition of CaCl_2_ was not required as the formation of calcium alginate was negligible. Pretreatment with a mixture of chloroform and methanol (1:1) is reported to reduce lipophilic contaminants [23]. The chloroform and methanol (1:1) extract is currently studied for its potential bioactive natural products. Pretreatment with 10% formaldehyde in ethanol is reported to reduce the polyphenolic contamination of polysaccharides [23]. Otherwise, the contamination could lead to unreliable observations when assessing the bioactivities of polysaccharides. Further, washing with 80% ethanol not only removes any remaining formaldehyde but also removes polar compounds, such as phenolic acids. The reduced levels of polyphenol, protein and ash content (a measure of mineral content) in polysaccharide fractions obtained by step gradient ethanol precipitation suggested the efficacy of the reported method. The current pretreatment and extraction strategy was developed based on observations by several previous studies and preliminary analysis [3,11,14,24].

Crude fucoidan is generally obtained by adding a large volume of ethanol to the water-based extract. Subsequent fractionation is done by filtering precipitated polysaccharides through a series of molecular weight cutoff membranes (ultrafiltration) or by chromatography using size exclusion or anion exchange columns [23]. Ultrafiltration and chromatography are not suitable for industrial-scale preparations considering the investment, cost of equipment and operation delays. The step gradient ethanol precipitation method offers convenience over conventional fucoidan purification techniques. However, the technique has not widely been used for fractionating fucoidans except for that in one reported study [7]. The gradient alcohol precipitation method is commonly used in dextran fractionation and less frequently for purifying other polysaccharides such as starch, hemicellulose, glucan, fructan, pectin, arabinan and pullulan [6]. Based on agarose gel electrophoresis, four different fractions with approximate molecular weight distributions of 230, 205, 90 and 60 kDa were successfully obtained using step gradient ethanol precipitation. The high yield of the SHC2 fraction suggested that most fucoidans in *S. horneri* had a molecular weight range of approximately 205 kDa. Furthermore, low molecular weight polysaccharide fractions indicated a higher degree of sulfation. This further relates to the solubility of polysaccharides, where the ionic characteristics of polysaccharides increase with an increased degree of sulfation. Hence, the step gradient ethanol precipitation method could be said to separate fucoidans based on both the molecular weight and solubility.

The structural features of the fucoidan heteropolysaccharide vary considerably depending on seaweed species and environmental conditions. These features include monosaccharide composition, connectivity, chain length, degree of sulfation and connectivity of sulfate groups. Due to its heterogeneous nature, a full-scale analysis of connectivity remains challenging. Based on the present observations, the fractions were identified as fucoidans by FTIR and chemical composition analysis. Though it is not a generally accepted explanation, we propose that fucoidans could be ranked based on their quality, with the major parameters including the amount of fucose and degree of sulfation. According to many studies, the fucose amount is directly proportional to the degree of sulfation, which suggests that most sulfate groups remain attached to fucose units in fucoidan [3]. The fucoidan fraction that had the most prominent antioxidant effects, SHC4, was found to contain 45.96 ± 0.36% polysaccharides, of which, 81.45% was fucose, which corresponded to 37.43% of the fraction weight with a sulfate content of 28.01 ± 0.50%. ^1^H NMR data revealed the presence of α-l-Fucp-(1→4) residues, which are a major feature of fucoidans. Hence, we designated SHC4 as a high-quality fucoidan. The lack of data on monosaccharide and sulfate group connectivity patterns is a limitation of the present study. The connectivity “methylation analysis” requires the fucoidan to be further fractionated, obtaining a less heterogeneous polymer mix.

Fucoidan is renowned for its potential antioxidant properties among a wide range of bioactivities [23]. The dose-dependent reduction of intracellular ROS levels in all fractions indicated the protective effect of fucoidans. Considering the slope observed for each fraction dose, the rate of the intracellular ROS reduction increased in succession, with the SHC4 fraction showing the most reduction. This suggested that low molecular weight and high-quality fucoidans had superior antioxidant properties. The increments observed for cell viability suggested that the protective effects of SHC4 were within the 25–100 µg mL^−1^ concentration range. The minor reduction in cell viability at 200 µg mL^−1^ concentration could be associated with toxic effects of sulfated polysaccharides under high concentrations. Further bioassays were conducted to assess the protective effects of SHC4 against UVB-induced oxidative stress and apoptosis. There was a reduction in UVB-induced apoptotic body formation, accumulation of Sub-G_1_ apoptotic cells and DNA damage, clarifying the potential antioxidant activity of SHC4. Further studies were then performed to understand the effects of SHC4 on the regulation of apoptosis.

The mitochondria-mediated apoptosis pathway is considered a major signaling route mediating apoptosis; the underlying mechanism can vary based on the type of cell, stimulus and other factors. Oxidative stress resulting from multiple factors, including the induction of UVB-radiation is reported to activate the mitochondria-mediated apoptosis pathway [18]. The pathway is initiated with the permeabilization of the mitochondrial outer membrane. The permeabilization is controlled by Bcl-2 family proteins, which includes anti-apoptotic proteins (Bcl-2 and Bcl-xL) and pro-apoptotic proteins (Bax, Bak, Bok, Bad, Bid, Bik, Bim, Bmf, Puma and Noxa). Their primary function is disrupting the functions of the Bcl-2 family proteins, thereby promoting the release of apoptogenic proteins present in the intermembrane space. Released apoptogenic proteins include cytochrome c, apoptosis-inducing factor (AIF) and endonuclease G. Cytochrome c with pro-caspase 9 and apoptosis protease activating factor (APAF-1) form an ‘apoptosome’. The apoptosome promotes caspase 9 activation, in turn activating effector caspases, which collectively execute apoptosis. Both AIF and endonuclease G contribute to DNA fragmentation and chromosomal condensation, features that are a hallmark of apoptosis [25]. Apart from the aforementioned mediators, permeabilization of the mitochondrial outer membrane causes the release of numerous proteins that antagonize the activation of caspases. Pro-apoptotic stimuli inducing p53 further aggravate mitochondrial pathway-mediated apoptosis. p53 plays a crucial role in ultraviolet-induced apoptosis in HaCaT keratinocytes [26]. Under physiological conditions, p53 is maintained at a low concentration, inhibiting its transcriptional activity. Activation and post-translational stabilization of p53 drives the expression of pro-apoptotic factors, provoking death pathways. Caspases, consisting of initiator and effector caspases, are proteases that mediate cell death. Initiator caspases, such as caspase-9, activate death receptors, whereas effector caspases directly mediate the cleavage of numerous cytoplasmic and nuclear substrates. Cytochrome c activates caspase-9 and subsequently activates downstream effector caspases such as caspase-3, −6 and −7 [9]. The cleavage of PARP, a nuclear enzyme that maintains DNA stability, repair and transcription, is another crucial feature of apoptosis. Effector caspases cause the cleavage of PARP, thereby inhibiting its catalytic activity [9]. The present observations clarified the effect of UVB on the initiation of mitochondria-mediated apoptosis proteins and the dose-dependent attenuation effects of SHC4. There could be numerous alternative pathways, other than the prominent mitochondria-mediated apoptosis that regulate oxidative stress-induced cell death. Hence, further studies could broaden the understanding of the effects of fucoidan on UVB-induced apoptosis.

Based on previous studies, fucoidans are reported to upregulate gene expression of HO-1 and SOD-1 in HaCaT keratinocytes via upregulating the transcription factor Nrf2 [21]. Similar results were observed during the present analysis, where SHC4 treatment caused a dose-dependent increase of HO-1 production in the cytosol and Nrf2 levels in the nucleus. This suggested that the protective effects of fucoidan against UVB-induced oxidative stress were mediated by Nrf2/HO-1 signaling.

## 5. Conclusions

Based on the present analysis, step gradient ethanol precipitation was identified as a desirable approach to obtain fucoidan fractions with different molecular weight distributions. The fucoidan fraction with a mean molecular weight of 60 kDa from *S. horneri* was mainly composed of α-L-Fucp-(1→4) linked fucose units with 37.43% fucose content and a 28.01 ± 0.50% sulfate content. The above fucoidan fraction, SHC4, demonstrated significant protective effects against UVB-induced photodamage in dermic keratinocytes by enhancing intracellular antioxidant defense. In the future, SHC4 could be studied for developing cosmetics with UV protective effects.

## Figures and Tables

**Table 1:** Yield and proximate composition of the major components in the fractions.

Yield (%)		SHC1	SHC2	SHC3	SHC4
		5.25	16.75	4.26	3.81
Sulfated polysaccharide content (%)	Polysaccharide	72.02 ± 0.46	62.29 ± 0.09	54.34 ± 0.00	45.96 ± 0.36
	Sulfate	13.85 ± 0.47	19.26 ± 0.11	23.64 ± 0.48	28.01 ± 0.50
Polyphenol content (%)		3.06 ± 0.44	2.59 ± 0.15	1.91 ± 0.30	1.24 ± 0.21
Protein content (%)		0.54 ± 0.03	0.68 ± 0.01	0.44 ± 0.02	0.50 ± 0.01
Ash content (%)		1.85 ± 0.11	0.88 ± 0.09	0.70 ± 0.12	0.54 ± 0.02

SHC1-SHC4 denote different polysaccharide fractions obtained via step gradient ethanol precipitation. Data represent the mean ± standard deviation of triplicate determinants (*n* = 3).
